# Pathogenic Parkinson’s disease mutations across the functional domains of LRRK2 alter the autophagic/lysosomal response to starvation^☆^

**DOI:** 10.1016/j.bbrc.2013.10.159

**Published:** 2013-11-29

**Authors:** Claudia Manzoni, Adamantios Mamais, Sybille Dihanich, Phillip McGoldrick, Michael J. Devine, Julia Zerle, Eleanna Kara, Jan-Willem Taanman, Daniel G. Healy, Jose-Felix Marti-Masso, Anthony H. Schapira, Helene Plun-Favreau, Sharon Tooze, John Hardy, Rina Bandopadhyay, Patrick A. Lewis

**Affiliations:** a Department of Molecular Neuroscience, UCL Institute of Neurology, Queen Square, London WC1N 3BG, UK; b Reta Lila Weston Institute and Queen Square Brain Bank, UCL Institute of Neurology, 1 Wakefield Street, London WC1N 1PJ, UK; c MRC Centre for Neuromuscular Disease, UCL Institute of Neurology, Queen Square, London WC1N 3BG, UK; d Helmholtz Zentrum München, GmbH Ingolstädter Landstraße 1, D-85764 Neuherberg, Germany; e Department of Clinical Neuroscience, UCL Institute of Neurology, Queen Square, London WC1N 3BG, UK; f Beaumont Hospital, 9 Beaumont Rd, Dublin 9, Co. Dublin, Ireland; g Hospital Donastia, San Sebastian, Spain; h London Research Institute, Cancer Research UK, London WC2A 3LY, UK; i School of Pharmacy, University of Reading, Whiteknights, Reading RG6 6AP, UK

**Keywords:** LRRK2, leucine rich repeat kinase 2, ROC, ras of complex proteins, COR, C-terminal of ROC, PD, Parkinson’s disease, ICC, Immunocytochemistry, LRRK2, Parkinson’s disease, Autophagy, Lysosomes, Signaling pathways

## Abstract

*LRRK2* is one of the most important genetic contributors to Parkinson’s disease (PD). Point mutations in this gene cause an autosomal dominant form of PD, but to date no cellular phenotype has been consistently linked with mutations in each of the functional domains (ROC, COR and Kinase) of the protein product of this gene. In this study, primary fibroblasts from individuals carrying pathogenic mutations in the three central domains of LRRK2 were assessed for alterations in the autophagy/lysosomal pathway using a combination of biochemical and cellular approaches. Mutations in all three domains resulted in alterations in markers for autophagy/lysosomal function compared to wild type cells. These data highlight the autophagy and lysosomal pathways as read outs for pathogenic LRRK2 function and as a marker for disease, and provide insight into the mechanisms linking LRRK2 function and mutations.

## Highlights

• Mutations in the ROC, COR and Kinase domain of LRRK2 alter the autophagic response to starvation.
• LC3-I/II ratio following starvation is altered by mutations, as well as p62 and WIPI2 positive puncta.
• This occurs independently of any alteration in downstream targets of mTORC1.

## 1 Introduction

Leucine Rich Repeat Kinase 2 (LRRK2) is a multidomain protein of unclear function containing two enzymatic domains, a GTPase (Ras of Complex Proteins, ROC) and a kinase, connected by a C-terminal of ROC (COR) domain and flanked by protein/protein interaction domains [1]. LRRK2 has been implicated in a number of cellular processes, including the control of neurite branching, synaptic vesicle recycling, macroautophagy (hereafter referred to as autophagy), protein synthesis through the mTOR pathway and mitochondrial function [2].

The central role of LRRK2 in Parkinson’s disease (PD) has been highlighted by the discovery of autosomal dominant mutations in *LRRK2* causing familial PD and the subsequent identification of the *LRRK2* locus as a risk factor for sporadic disease [3,4]. A key question regarding the role of autosomal dominant coding change mutations in PD is what the cellular consequences of these mutations are, and how they lead to disease [2]. All known highly penetrant missense mutations (N1437H, R1441C, R1441G, Y1699C, I2012T, G2019S, I2020T) are located in the enzymatic core of LRRK2 – the ROC/COR/kinase triad [4], leading to a number of studies examining the impact of mutations on the enzymatic activities of this protein. The G2019S mutation, the most common disease linked variant in LRRK2 in Europeans, has been consistently associated with increased kinase activity, and mutations in the ROC and COR domains display reduced GTPase activity [5–9]. However, no single biochemical phenotype has been linked to mutations in all three of these domains. The only reported cellular phenotype that consistently correlates with penetrant mutations is cytotoxicity, which is dependent upon kinase activity [10–12].

A major gap in our understanding of LRRK2 pathobiology is, therefore, a biological phenotype for LRRK2 that correlates with disease genotype. The identification of a phenotype common to all pathogenic mutations would provide a target for drug testing in a manner analogous to the use of Aβ42 levels in Alzheimer therapies [13,14]. Moreover, it would indicate a specific pathway to be studied to find markers for early diagnosis of disease, an urgent unmet need for PD [15].

A number of studies have highlighted a putative role for LRRK2 in the regulation of the autophagy/lysosomal pathway. Several groups have reported an impact of the common G2019S mutation (located in the kinase domain of LRRK2) on either basal or induced autophagy [16–21]. Mutations in the ROC domain of LRRK2 have also been reported to alter autophagy in human and yeast models for LRRK2 [17,22]. With regard to the physiological function of LRRK2, both knockout, knockdown and chemical inhibition of LRRK2 have been reported to alter autophagy [17,23,24]. Based on these reports, human fibroblasts carrying mutations across the enzymatic core of LRRK2 were compared to wild type cells in order to test whether there is a consistent impact of these mutations upon the autophagy/lysosomal pathway.

## 2 Materials and methods

### 2.1 Antibodies

Antibodies used were as follows: rabbit LC3 antibody (NB100-2220, Novus Biologicals); LRRK2 antibodies (3514-1, Epitomics); total S6 antibody (2317, Cell Signaling); phospho Ser235/236 S6 antibody (2211S, Cell Signaling); total P70S6K antibody (sc-8418, Santa Cruz); phospho Thr389 P70S6K (sc-11759, Santa Cruz); total 4EBP1 (81149, Santa Cruz); phospho Ser65 4EBP1 (9451S, Cell Signaling); mouse p62 antibody (610833, BD Transduction Labs); rabbit p62 antibody (BML-PW9860-0025, Enzo Life Sciences); mouse WIPI2 antibody (kindly supplied by Dr. S.Tooze); mouse LAMP1 (H4A3, Abcam) and mouse β-actin antibody (A1978, Sigma Aldrich).

### 2.2 Cell culture and Western blotting

Fibroblasts were grown in DMEM containing 10% FCS. Fibroblasts were isolated from genetically defined individuals following local ethical approval and full informed consent (see Ref. [25] for details). mTOR inhibition was achieved by overnight (16 h) serum deprivation followed by substitution of the medium with Earle’s balanced salts solution for 2 h. Re-activation of the mTOR pathway was obtained by MEM non-essential amino acid supplement added for 30 min to the fibroblast culture after starvation. Cells were collected in DPBS; the cell pellet was washed once and then lysed in a buffer containing: 0.5% Triton X-100, 2 mM EDTA, 150 mM NaCl, 0.5% sodium deoxycholate, 0.1% SDS, protease inhibitors (cOmplete, Roche) and phosphatase inhibitors (Halt, Pierce) in 50 mM TRIS–HCl pH 7.5. Cell lysates were frozen immediately upon collection. Following thawing, lysates were clarified by centrifugation at 10000*g* for 5 min at 4 °C and the protein concentration was assessed by BCA assay (BCA Protein Assay Kit, Pierce). 10 μg aliquots were prepared, denatured in NuPAGE sample buffer (Invitrogen) for 10 min at 70 °C and analysed by immunoblot as previously described [24]. Statistical analyses were performed by the use of the Prism software (GraphPad) as described in the text.

### 2.3 Immunocytochemistry

Cells were plated and analysed by ICC as previously described [24]. For the quantification experiments, images were acquired with a Leica CTR 6000 fluorescence microscope, and processed by the LAS AF Lite software. Cell counts for WIPI2 puncta or p62 immunoreactivity were performed on the acquired images manually by a blinded operator using the cell counter plugin tool in the ImageJ software package. Graphs and statistical analyses were performed using Prism software.

## 3 Results

**Fig. 1:**
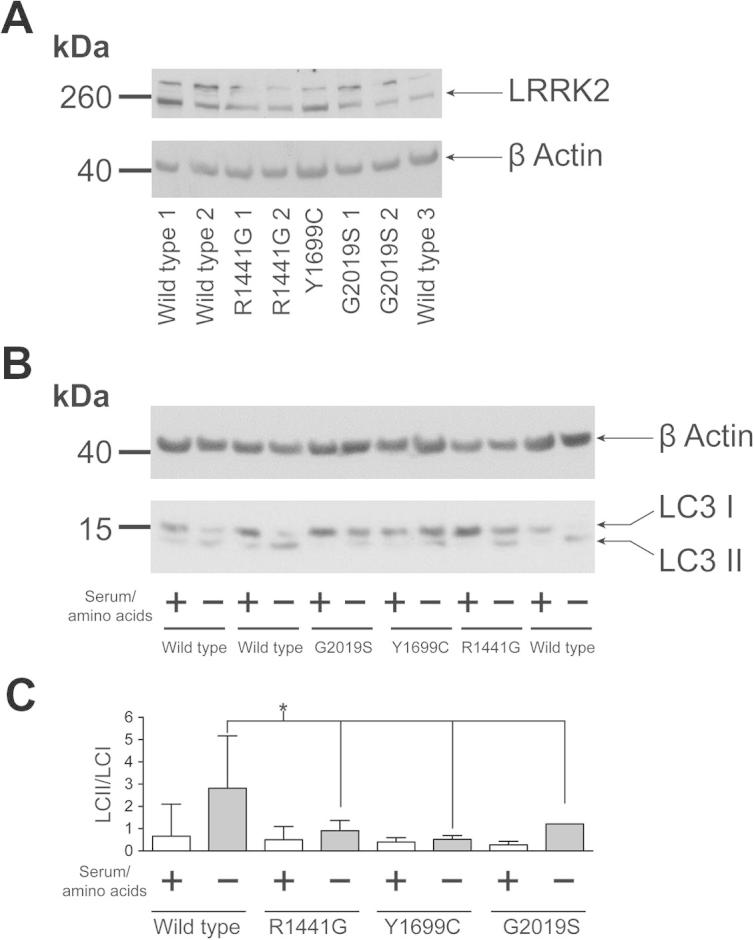
LC3-II/LC3-I ratio after starvation is altered in fibroblasts carrying LRRK2 mutations. (A) Immunoblot analysis reveals that LRRK2 is expressed at equivalent levels in the fibroblast cells used in this study. (B) Under normal growing conditions (+) no differences between genotypes are observed in the ratio of LC3-I to LC3-II, with LC3-I the most abundant form. After starvation from serum and aminoacids (–), LC3-II increases and LC3-I decreases in wild type fibroblasts thus leading, as expected, to an overall increase in the LC3-II/LC3-I ratio, shown in (C). For all the mutant fibroblasts after starvation, the increase in LC3-II and the concomitant decrease in LC3-I is less evident thus leading to a significantly smaller increase in the LC3-II/LC3-I ratio when compared with starved wild type cells (one-way ANOVA, Dunnett’s test, * *p* < 0.05). The gel is representative of 6 independent experiments, with data pooled to generate the graph in which means and standard deviations are shown. 3 different wild type lines, 2 different R1441G, 2 different G2019S and 1 Y1699C mutant lines have been used.

The impact of mutations in the three central domains of LRRK2 upon autophagy and lysosomal function were assessed in patient derived fibroblasts harboring LRRK2 mutations – the R1441G mutation in the ROC/GTPase domain, the Y1699C mutation in the COR domain and the G2019S mutation in the kinase domain – and age/sex matches control cells [25]. Levels of LRRK2 protein were comparable in wild type and mutation carrying cells (Fig. 1A).

**Supplementary Figure 1:**
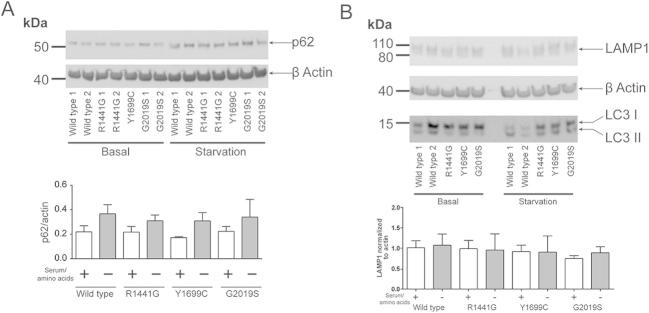
No differences in p62 or LAMP1 levels observed between wild type and mutant cells. Wild type and mutant fibroblasts were assessed under normal growing conditions (+) and after serum and aminoacids starvation (−). Total levels of p62 (A) and LAMP1 (B) are identical for all the fibroblasts analysed. 2 different wild type lines, 2 different R1441G, 2 different G2019S and 1 Y1699C mutant lines have been used.

**Fig. 2:**
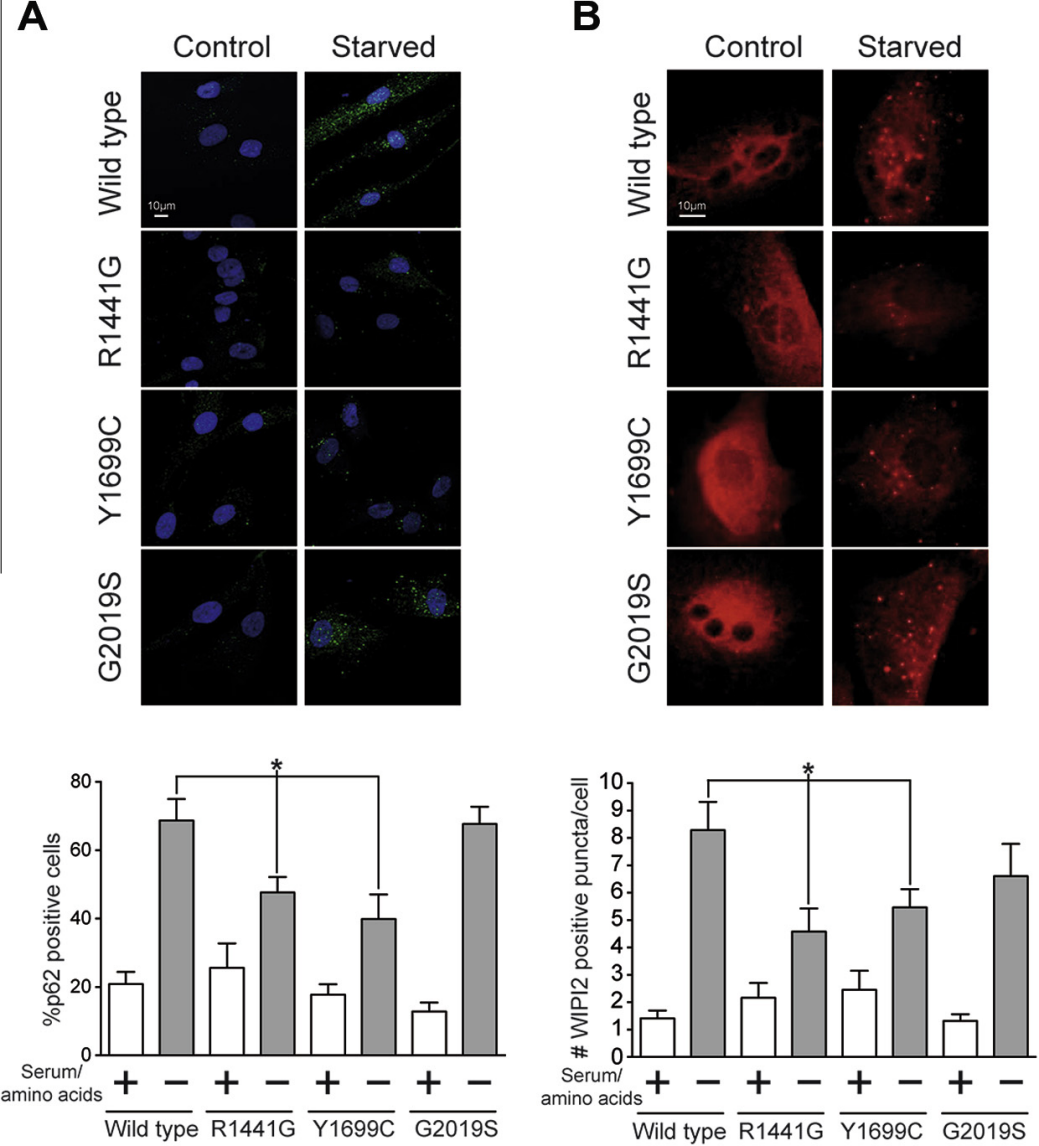
Immunocytochemistry reveals alterations in the amount of p62 positive cells and WIPI2 puncta in starved fibroblasts carrying LRRK2 mutations compared to wild type cells. The percentage of p62 positive cells (A) and the amount of WIPI2 puncta per cells (B) have been assessed under basal and starved growing conditions. The percentage of p62 positive cells and the amount of WIPI2 puncta per cells always increase after starvation indicating an activation of autophagy. The increases observed for R1441G and Y1699C mutation carriers are significantly smaller than those observed for the wild type cells. No differences were observed for the G2019S fibroblasts (one-way ANOVA, Dunnett’s post hoc test using starved wild type as reference). WIPI2 puncta: an average of 22 frames has been acquired at 60X magnification for each cell type in control and starvation reaching a total number of counted cells as follows: 131, wild type control; 152 wild type starved; 97 G2019S control, 117 G2019S starved; 36 Y1699C control, 78 Y1699C starved; 73 R1441G control, 75 R1441G starved. P62 positive cells: for every sample 5 different frames from 2 independent slides have been acquired at 40x magnification. The total number of counted cells was as follows: 344, wild type control, 314 wild type starved; 150 G2019S control, 198 G2019S starved; 170 Y1699C control, 117 Y1699C starved; 355 R1441G control, 318 R1441G starved.

**Fig. 3:**
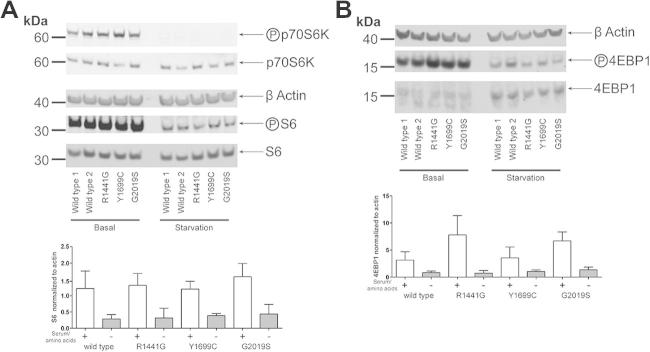
mTORC1 pathway phospho-state is not altered by mutations in LRRK2. Wild type and mutant fibroblasts were assessed under normal growing conditions (+) and after serum and aminoacids starvation (−). Total levels of S6/p70S6K (A) and 4EBP1 (B) are identical for all the fibroblasts analysed. No differences in phosphorylation are observed between genotypes under either condition. 2 different wild type lines, 2 different R1441G, 2 different G2019S and one Y1699C mutant lines have been used.

To investigate autophagy pathways in the presence and absence of mutations in LRRK2, the autophagic marker LC3 was examined under normal growth conditions and following starvation from serum and amino acids (Fig. 1B and C). No differences were observed in LC3 processing (as measured by conversion of LC3-I, the cytosolic form of LC3, into the vesicle associated LC3-II form of the protein) between genotypes under normal growth conditions. Under starvation conditions, a significant difference was observed between wild type and mutant cells in the ratio between LC3-I and LC3-II, suggesting that mutations in LRRK2 disrupt the autophagic response to starvation. It should be noted that no significant alteration in the ratio of LC3-II to β-actin was observed between genotypes (data not shown), suggesting that the impact of LRRK2 mutations upon starvation-induced autophagy is more complex than a straightforward increase in bulk autophagy. This was further emphasized by the absence of detectable changes in p62 levels when analyzed by immunoblot after starvation across wild type and mutant fibroblasts or in levels of LAMP1, a marker for the lysosomes (Supplemental Fig. 1). To investigate the autophagic response of LRRK2 mutation carrying cells using an alternative technique, autophagic vesicle formation was assessed by ICC under normal growing and starvation conditions. Using the vesicular marker WIPI2 and p62, a significant difference in p62 positive cells and in WIPI2 positive puncta per cell were observed for the R1441G and Y1699C mutations compared to wild type cells under starvation conditions (see Fig. 2). No differences were observed between wild type and G2019S mutation cells. To establish whether these alterations were accompanied by changes in mTORC1 signaling, a key regulator of autophagy and translational repression, the phosphorylation states of p70S6K, S6 and 4EBP1 were evaluated (Fig. 3A and B). No differences were observed between genotypes under either normal growing conditions or following starvation.

## 4 Discussion

Mutations in LRRK2 cause an autosomal dominant form of PD that is almost indistinguishable from the idiopathic form of the disease. Since 2004, a number of cellular processes have been linked to the function of LRRK2, however no cellular phenotype has been found to be altered in the same way by penetrant mutations in each of the three central domains of LRRK2. This has left a major gap in our understanding of the disease process deriving from these mutations. The results from this study demonstrate that three penetrant mutations in the ROC, COR and kinase domains of LRRK2 result in an altered cellular response to starvation conditions as reflected in altered pools of cytosolic and membrane associated LC3. These data support a pathogenic mechanism consistent with alterations in the autophagy-lysosomal pathway and in general vesicle metabolism, with a possible mechanistic dichotomy between mutations in the ROC-COR and kinase domains. Although the exact mechanism(s) whereby LRRK2 impacts on LC3 processing is unclear, these data echo, in a physiological cellular system, previous reports from overexpression model systems implicating LRRK2 in the cellular process of autophagy and lysosomal degradation [16,17,19,21,22].

Two previous studies have highlighted alterations in autophagy associated with the G2019S mutation at an endogenous level [18,20]. In contrast to data in the current study, where a difference between wild type and mutant cells is only observed under starvation conditions, Bravo-San Pedro and colleagues observed an alteration in basal autophagy in fibroblasts from individuals carrying the G2019S mutation. The most likely explanation for the different results obtained in these two studies is that the culturing conditions used to examine basal autophagy by Bravo-San Pedro et al. are significantly different to those used in the study presented above. Sanchez-Danes and co-workers reported altered autophagy in neurons differentiated from induced pluripotent stem cells that had been derived from patients carrying the G2019S mutation, suggesting that changes in the regulation of autophagy due to mutations in LRRK2 are a feature of multiple cell types, including cells impacted in Parkinson’s disease. In contrast to Bravo-San Pedro (and similar to the data in this study), Sanchez-Danes et al. did not observe an alteration in basal autophagy in the G2019S mutation fibroblasts from which the pluripotent cells they used in their study were derived.

An important insight arising from the data reported above is the absence of any alteration in the regulation of downstream translational targets of mTORC1 (either S6 or 4EBP1). This is in contrast to a study in *Drosophila melanogaster* which suggested that LRRK2 regulated the phosphorylation of 4EBP1 [26], but is congruent with recent studies that did not observe any interaction between LRRK2 and 4EBP1 either *in vitro* [27], or in mouse models or human brain tissue [28].

Examining the catalytic domain triad of LRRK2, the ROC and COR domains can be viewed as distinct from the kinase domain from a functional, structural and evolutionary perspective [29]. The G2019S mutation, within the kinase domain, increases the kinase activity of LRRK2; mutations in the GTPase (R1441G) and in the COR (Y1699C) domains are linked to a decrease in the GTPase activity. The data in this study demonstrate that mutations in the ROC-COR domains alter the ability of the cell to respond to starvation impairing the physiological increase in the concentration of WIPI2 and p62 positive vesicles upon starvation. This was paired by a dysregulation in the distribution between the membrane bound and the cytosolic forms of LC3 thus suggesting that the autophagy/lysosomal pathway may be potentially affected by the R1441G and Y1699C mutations in LRRK2. The G2019S mutation produced the same alteration in the metabolism of LC3 in the absence of concomitant changes in vesicle content during starvation, suggesting that the precise route to dysfunction may be dependent upon which of LRRK2s enzymatic activities is impacted by mutation. Given the complex relationship between the enzymatic activities of LRRK2, and the recent observation that the G2385R polymorphism linked to disease risk in Asian populations acts to decrease kinase activity [30], it is possible that disruption of enzymatic function *per se* is the key biochemical read out of LRRK2 dysfunction in PD – with distinct but convergent phenotypic consequences. An important test of this will be examining the impact of the G2385R polymorphism on autophagic response. The precise mechanism whereby LRRK2 regulates autophagy remains to be determined, however the data in this study and from studies focusing on chemical inhibition of LRRK2 kinase activity [24] suggest that this may be *via* a non-canonical autophagy pathway, independent of mTORC1.

The observation that alterations in the autophagic lysosomal pathway in response to starvation are common cellular feature of LRRK2 mutations is important for several reasons. First, this reiterates the importance of the autophagy/lysosomal pathway to neurodegeneration, and in particular a role of this pathway in PD [31,32]. The links between vesicle metabolism, lysosomes, autophagy and protein misfolding diseases provide a plausible connection between the cellular phenotype observed in this study, and the pathogenesis and pathology of LRRK2 PD. Secondly, and independent of the actual role in the pathological pathways leading to disease in LRRK2 cases, this cellular phenotype provides a read out for LRRK2 dysfunction. This may be amenable to screening of compounds directed against the pathogenic impact of LRRK2 mutations, thereby providing a valuable tool in the urgent search for modifiers of PD disease progression and early markers of disease. This study also re-emphasizes the importance of studying a range of mutations in LRRK2, rather than focusing on the most common G2019S mutation. Finally, in the context of the role that LRRK2 plays in human disease, it is noteworthy that LRRK2 has been implicated in a number of human disorders, including Crohn’s disease, Cancer and Leprosy in addition to PD [33]. The autophagy/lysosomal pathway has been linked to all of these disorders, and the observation that LRRK2 is intimately involved in this cellular process may be relevant to its role numerous human conditions, further emphasising the need to clarify the mechanisms that link LRRK2 and the autophagy-lysosomal pathway.
